# A retrospective assessment of different endodontic treatment protocols

**DOI:** 10.7717/peerj.8495

**Published:** 2020-01-30

**Authors:** Andreas Bartols, Carsten Bormann, Luisa Werner, Melanie Schienle, Winfried Walther, Christof E. Dörfer

**Affiliations:** 1 Dental Academy for Continuing Professional Development Karlsruhe, Karlsruhe, Germany; 2 Clinic for Conservative Dentistry and Periodontology, School for Dental Medicine, Christian-Albrechts-University, Kiel, Germany; 3 Chair of Econometrics and Statistics, Karlsruhe Institute of Technology, Karlsruhe, Germany

**Keywords:** Non-surgical endodontic treatment, Root canal treatment, Endodontic outcome, Stainless steel hand files, Rotary NiTi multiple files, Single files, Reciproc

## Abstract

**Background:**

The aim of this study was to assess the clinical impact of non-surgical root canal treatments (NSRCT) performed with different treatment protocols on the probability of tooth survival without untoward events and to identify predictors influencing the outcome.

**Methods:**

During the period from July 1999 to October 2016, 5,858 patients were identified in which 9,967 NSRCTs were performed. The treatments were followed up and divided into three groups. In Group 1 root canal treatment was performed with hand instruments, in Group 2 with multiple file rotary instruments and passive ultrasonic irrigation (PUI), and Group 3 was treated with Reciproc instruments and PUI. Untoward events were defined as orthograde retreatment, apicoectomy or extraction of the tooth after initial treatment. Weibull regression was used to analyse the data.

**Results:**

A total of 9,938 cases could be included into the analyses. The results showed 5-years predicted survival rates without untoward events of 73.9% (95% CI [71.7%–76.1%]), 75.1% (95% CI [71.7%–78.0%]) and 78.4% (95% CI [75.1%–81.4%]) for study group 1 (*N* = 5,580), 2 (*N* = 1,700) and 3 (*N* = 2,658), respectively. The differences between Group 1 and 3 were statistically significant (*p* < 0.006). Higher age of the patient (per year increase) and number of earlier NSRCTs (per unit increase) reduce the survival without untoward events statistically significant (both *p* < 0.02), while treatment of premolars had a statistically significant lower hazard ratio [0.89 (95% CI [0.79–0.99]; *p* = 0.030)] compared to treatment of molars and anterior teeth. A higher number of supportive periodontal treatments (per unit increase) improved tooth survival without untoward events highly significant (*p* < 0.0001).

**Discussion:**

More recent endodontic treatment protocols involving reciprocating instruments and PUI appear to be associated with higher tooth survival rates without untoward events compared to hand instruments.

## Introduction

Tooth survival rates following endodontic treatment are apparently constant since decades (Friedman & Mor, 2004). At least there is no systematically documented improvement, by means of increasing success of endodontic treatment or tooth survival rates over time (Ng et al., 2007). This is in contrast to the considerable efforts that have been undertaken during the same time period to develop new tools and endodontic techniques that would facilitate the treatment procedure and ideally improve the outcome of endodontic treatment.

The methodological and technological changes aimed to improve endodontic treatment outcome can be categorized into two main fields. One field of technological progress has certainly been the root canal preparation itself, accompanied by the advancement of appropriate tools from stainless steel hand files (HF) (Ingle, 1961) through rotary nickel-titanium (NiTiR) multiple-file (MF) instruments (Thompson & Dummer, 1997) to machine driven single-file reciprocating root canal instruments (SF) (Bartols, 2013). Another field of progress is characterized by the establishment of protocols for efficient intracanal disinfection starting from syringe-irrigation with sodium hypochlorite (NaOCl) to passive ultrasonic irrigation (PUI) (Jensen et al., 1999).

Since HFs have been the method of choice for many years, data on long term outcome of endodontic treatment are available (Kerekes & Tronstad, 1979; Orstavik, Kerekes & Eriksen, 1987; Weiger, Rosendahl & Lost, 2000). Most of these studies, regardless if recent or not (Friedman & Mor, 2004), have shown comparable high success or tooth survival rates. For newer techniques of endodontic treatment, such as the use of NiTiR instruments, the data available are more limited (Koch et al., 2015; Ng, Mann & Gulabivala, 2011a). Finally, for the most modern techniques for endodontic treatment, i.e., using reciprocating instruments, to our knowledge, no studies have been published that evaluate long-term success or tooth survival as clinically relevant outcome. Recently, both other workgroups and ours have demonstrated that the short-term objective of pain reduction can be similarly achieved by using either HF, MF or SF endodontic instruments (Bartols, Laux & Walther, 2016a; Bartols et al., 2016b; Kherlakian et al., 2016; Relvas et al., 2015). However, this short-term treatment outcome by no means reflects the ultimate treatment-dependent biological objective(s) of preserving or regaining periapical sound tissues or of survival of the tooth treated.

The success of root canal treatment can be assessed by applying either strict or more lenient criteria. The strict definition of endodontic success includes the maintenance of radiographically sound periapical tissues and additionally clinical normalcy without signs and symptoms of disease. In case of a pre-existing apical periodontitis the healing of the lesion should at least be initiated and ideally resolved through endodontic therapy (Friedman & Mor, 2004). A more lenient definition of treatment success in root canal treatment is the pain-free functional survival of the endodontically-treated tooth (Friedman & Mor, 2004). Surely, survival as a clear dichotomous variable is an outcome measure that is not prone to subjectivity and interpretation as radiographic outcome measures are. Therefore, the latter definition of treatment success simplifies analyses and is more appropriate when dealing with large-scale retrospective clinical or claims data.

In the present study we sought to assess the clinical impact of technological changes in non-surgical root canal treatments (NSRCT)—performed under routine care conditions—on the probability of tooth survival without untoward events. Moreover, we aimed to identify additional putative predictors influencing outcome of endodontic therapy.

## Methods

A data base of own claims data from the outpatient clinic of the Dental Academy of Continuing Professional Development Karlsruhe, Germany was used. All data were anonymized and extracted for analyses without reference to patients. Because of the retrospective character of data collection, the study was a non-intervention clinical trial and did not interfere with the psychological or physical integrity of patients. The study was conducted in conformity with the Declaration of Helsinki and according to the Professional Code for Physicians of the Medical Council of the State of Schleswig-Holstein. The Institutional Review Board of the University of Kiel gave the ethical approval (D515/17).

### Database and study sample construction

For this retrospective study all NSRCT cases that were performed from July 1999 to October 2016 were included. Endodontic treatments on deciduous teeth were excluded. Further inclusion or exclusion criteria were not defined.

An anonymized copy of the invoicing database of the practice administration software (EVIDENT, Bad Kreuznach, Germany) was created for the data analyses of the present study. An interface was programmed for this, which enabled the creation of a tooth history by means of a database query. In the database, all treatments performed on a tooth are coded in accordance with the “BEMA tariff” (KZBV, 2019) of the statutory health insurance companies in Germany, supplemented by invoice codes in accordance with the tariff of private fees for dentists (Gebührenordnung für Zahnärzte “GOZ” (BZÄK, 2019) in Germany. Thereby it was possible to evaluate the services provided independently of the insured person’s status. The invoice codes for vital extirpation “VitE” and trepanation “Trep1” were used as index variables for case identification. A plausibility check was then carried out via the following invoice code “WK” (root canal preparation) to determine whether the tooth was actually subsequently endodontically treated. After that, a history was created by database query for each identified case with all subsequent invoice codes with the corresponding date of service delivery up to the last available dentist/patient contact. The time of the root filling (invoice code “WF”) defined the starting point of the observation. Excluded from the observation were cases in which the therapy was not completed with the root canal filling (code “WF”) or in which an apicoectomy (invoice codes “WR1”, “WR2” or “WR3”) was performed simultaneously with the code “WF”. The end of the observation was defined as either the date of tooth removal (invoice codes “X1”, “X2”, “X3”, “Ost1” and “Ost2”), the date of retreatment after index treatment (recurrence of codes “WK” and/or “WF” on the same tooth), the date of apicoectomy (later occurrence of codes “WR1”, “WR2” or “WR3”) in the case history or the date of the last dentist/patient contact. The various treatment methods were identified using the invoice code “Phys” for PUI. All treatments without this code were performed with HF (variable “Group 1”) and all treatments up to October 2011 were performed with MF Race/BioRace instruments (FKG, La Chaux de Fonds, Switzerland) supplemented with PUI (variable “Group 2”) if the invoice code “Phys” was accounted for in conjunction with the index variables. All therapies from November 2011 onwards were performed exclusively with SF Reciproc instruments (VDW, Munich, Germany) supplemented with PUI (variable “Group 3”). In addition, the tooth code (FDI two-digit notation), the date of birth and the variable “gender” were extracted directly from the copied original database. The categorical variables “root canal filling at the same appointment as canal preparation” (if “WK” date = “WF” date then “yes”), “tooth treated = vital” (if initial treatment = “VitE” then “yes”) and “type of tooth” (”anterior” = FDI Code 13 or 12 or 11 or 21 or 22 or 23 or 33 or 32 or 31 or 41 or 42 or 43; “premolar” = FDI Code 14 or 15 or 24 or 25 or 34 or 35 or 44 or 45; “molar” = FDI Code 16 or 17 or 18 or 26 or 27 or 28 or 36 or 37 or 38 or 46 or 47 or 48) were transformed within the table from the extracted data. In addition, “number of supportive periodontal treatments” (code “1040”), “number of intracanal dressings” (code “Med”) on the same tooth and “number of earlier NSRCTs” as well as “number of earlier surgical endodontic treatments” on the same patient were searched from the respective tooth histories and introduced as variables. The variable “participants’ age” at the time of endodontic treatment was calculated from the difference between the date of “WF” and date of birth. All variables were then collected in an Excel-table (Microsoft, Redmont, USA) for further processing in the statistical program.

### Endodontic technologies—study groups

The study groups reflect the technological changes in root canal treatment over the years. From 1999 on, the following treatment protocols have been performed:

#### Group 1: Hand instrumentation (*N* = 5,580)

From July 1999 to 2007 endodontic treatments have been performed with stainless steel hand instruments (K-files) using the standardized technique (Kerekes & Tronstad, 1979). A size of at least ISO 35 has been the goal of apical root canal enlargement. All root canals were disinfected with 3.5% NaOCl. Canal irrigation has been performed with syringes and needles (both Braun, Melsungen, Germany). The working length was determined radiographically and for root canal fillings gutta-percha and AH Plus (Dentsply, Konstanz, Germany) were used with cold lateral compaction.

#### Group 2: Multiple file rotary instruments and PUI (*N* = 1,700)

In 2008, rotary NiTi instruments and PUI were additionally introduced into the outpatient clinic. RaCe (FKG, La Chaux de Fonds, Switzerland) and BioRaCe instruments (FKG, La Chaux de Fonds, Switzerland) have been used for endodontic treatments of this group. A manual glide path preparation with stainless steel hand-instruments up to a #15 file preceded all rotary preparations. Working length was determined by an apex locator and radiographically. Irrigation solutions included 3.5% NaOCl and 15% citric-acid. Occasionally, 2% chlorhexidine was also added. PUI was always applied after final mechanical preparation of the root canal. Therefore, fresh NaOCl was placed into the root canal and subsequently either a spreader was activated with ultrasonic energy or an IRRI-K file (Acteon, Mettmann, Germany) was used with an ultrasonic device. The root canals were filled with cold lateral compaction of Gutta-percha and AH Plus.

#### Group 3: Single file reciprocating instruments and PUI (*N* = 2,658)

Starting in November 2011, endodontic treatments were exclusively performed using Reciproc instruments (VDW, Munich, Germany), following the manufacturer’s instructions for glide path free preparation (Yared, 2011). Only in rare cases a manual glide path preparation was performed, that means, in cases where Reciproc instruments were not able to prepare the complete root canal to working length. Working length was determined by an apex locator and/or radiographically. PUI was always applied for root canal disinfection. 18% Ethylenediaminetetraacetic acid (EDTA) was used for smear layer removal and 3.5% NaOCl for root canal disinfection. The root canal obturation has been performed either with lateral compaction or warm vertical compaction with Gutta-percha and AH plus (Dentsply, Konstanz, Germany).

### Operators

All endodontic treatments were performed by approved dentists with a corresponding degree. None of the operators had the formal specialist designation of an endodontist and none had a special training in the sense of a university-based postgraduate endodontic residency or master program.

### Outcome measures

We specified tooth survival without untoward events as main outcome parameter that was defined as the retention of the tooth observed without endodontic retreatment or apicoectomy during the observation period. Consequently, this parameter reflects a patient relevant outcome measure of the first intervention that was assessed for all cases in the follow-up period after initial treatment. The aforementioned covariates (see variables in “*Database and study sample construction*”) were assessed to elucidate factors that affect treatment outcome.

### Statistical analyses

R (Version 3.4.0. Win x64, The R-Foundation, Vienna, Austria) was used for all statistical analyses. A Weibull regression analysis (Zhang, 2016) was performed to take potential factors into account that affect survival. Significance was evaluated with the help of p-values with admissible *α*-type error of 0.05. Moreover hazard ratios (HR) were calculated according to the publication of Zhang (2016). The 1-, 2-, 3-, 4- and 5-years survival probability for each of the three groups was estimated on the basis of the regression results, whereby all continuous covariates were set to the corresponding mean value and all categorical variables were set to the lowest level. The results of the estimation were the time points for the quantiles of the occurrence of an event. Then the quantile q was searched for, for which the time amounts the corresponding observation year and then the survival probability after 1-, 2-, 3-, 4- and 5 years was calculated from 1-q for each group.

## Results

In our database 5858 patients were identified in which 9967 NSRCTs were performed during the period from July 1999 to October 2016. 9,920 cases (Group 1: *N* = 5,580, Group 2: *N* = 1,700, Group 3: *N* = 2,658) could be included into the regression analysis. Two cases had to be excluded because the data sets were implausible (date of retreatment was after the date of extraction), 27 cases had to be excluded because the treated teeth were deciduous teeth and 18 cases were not considered in the regression analysis due to missing data. Detailed characteristics of the included cases are summarized in Table 1. Moreover, 49 general dentists were identified that worked from July 1999 to October 2016 in the outpatient clinic and provided endodontic treatments.

**Table 1:** Characteristics of included treatments.

		**Study group**			**Significance**
	Total	Group 1	Group 2	Group 3	*P*-value
Included patients *N*	**5,858**^†^	3,489	1,304	1,953	
Included endodontic treatments *N*	**9,938**	5,580	1,700	2,658	
Mean No. of treatments per patient (SD)	**1.70 (1.12)**	1.60 (1.01)^a^	1.31 (0.64)^b^	1.36 (0.75)^b^	ANOVA *F* = 76.101, *p* < 0.001
Mean age of patient at the time of included treatment (SD)	**50.8 (17.1)**	50.2 (17.0)^a^	50.5 (17.2)^a^	52.0 (17.2)^b^	ANOVA *F* = 9.629, *p* < 0.001
					
Mean time of observation in years (min. - max.)	**3.9 (0.0–17.6)**	5.2 (0.0–17.6)	3.4 (0.0–8.2)	1.6 (0.0–4.8)	
Events during observation period					
Retreatments *N* (%)	**313 (3.1)**^‡^	263 (4.7)	29 (1.7)	21 (0.8)	
Root end surgeries *N* (%)	**259 (2.6)**^‡^	194 (3.5)	44 (2.6)	21 (0.8)	
Extractions *N* (%)	**1,738 (17.5)**^‡^	1,328 (23.8)	239 (14.1)	171 (6.4)	
Combined to untoward events *N* (%)	**2,140 (21.5)**^‡^	1,635 (29.3)	295 (17.4)	210 (7.9)	
					
Gender (female) *N* (%)	**4,977 (50.2)**	2,753 (49.4)	844 (49.7)	1,380 (52.0)	Ch*i*^2^ = 5.076, *p*= 0.079
Type of tooth					
Anterior teeth *N* (%)	**2,664 (26.8)**	1,549 (27.8)^a^	418 (24.6)^b^	697 (26.2)^a,b^	
Premolars *N* (%)	**3,046 (30.7)**	1,723 (30.9)^a^	530 (31.2)^a^	793 (29.8)^a^	
Molars *N* (%)	**4,228 (42.5)**	2,308 (41.4)^a^	752 (44.2)^a^	1,168 (43.9)^a^	Chi^2^ = 10.374, *p*= 0.035
Sensitivity = vital *N* (%)	**4,500 (45.3)**	2,637(47.3)^a^	800 (47.1)^a^	1,063 (40.0)^b^	Chi^2^ = 40.975, *p* < 0.001
Root canal filling in the same appointment as canal preparation *N* (%)	**1,503 (14.1)**	737 (13.2)^a^	95 (5.6)^b^	671 (25.2)^c^	Chi^2^ = 348.472, *p* < 0.001
Total *N* (%)	**9,938 (100.0)**	5,580 (100.0)	1,700 (100.0)	2,658 (100.0)	
					
Mean No. (SD) of supportive periodontal treatments in the tooth treated	**1.66 (3.05)**	1.42 (3.13)^a^	2.50 (3.56)^b^	1.64 (2.35)^c^	ANOVA *F* = 83.878, *p* < 0.001
Mean No. (SD) of intracanal dressings	**1.59 (1.26)**	1.83 (1.43)^a^	1.64 (0.95)^b^	1.06 (0.82)^c^	ANOVA *F* = 354.528, *p* < 0.001
Mean No. (SD) of earlier NSRCTs in the same patient	**0.96 (1.61)**	0.83 (1.44)^a^	1.04 (1.65)^b^	1.17 (1.88)^c^	ANOVA *F* = 44.135, *p* < 0.001
Mean No. (SD) of earlier surgical endondontic treatments in the same patient	**0.03 (0.25)**	0.02 (0.22)^a^	0.04 (0.30)^a,b^	0.04 (0.30)^b^	ANOVA *F* = 6.554, *p*= 0.001

**Notes.**

† The sum of the number of patients in each group does not correspond to the total number of patients, as some patients were treated with different methods on different teeth.

‡ A statistical comparison was not conducted due to the different observation periods.

Different superscript letters in the same row show statistically significant differences between the different groups with *p* < 0.05.

In cases with Chi^2^-Tests, post hoc analyses involved pairwise comparisons using the *z*-test of two proportions with a Bonferroni correction. In cases with ANOVA, post hoc analyses were performed with Tukey-HSD-test.

NSRCT non-surgical root canal treatment

**Figure 1:**
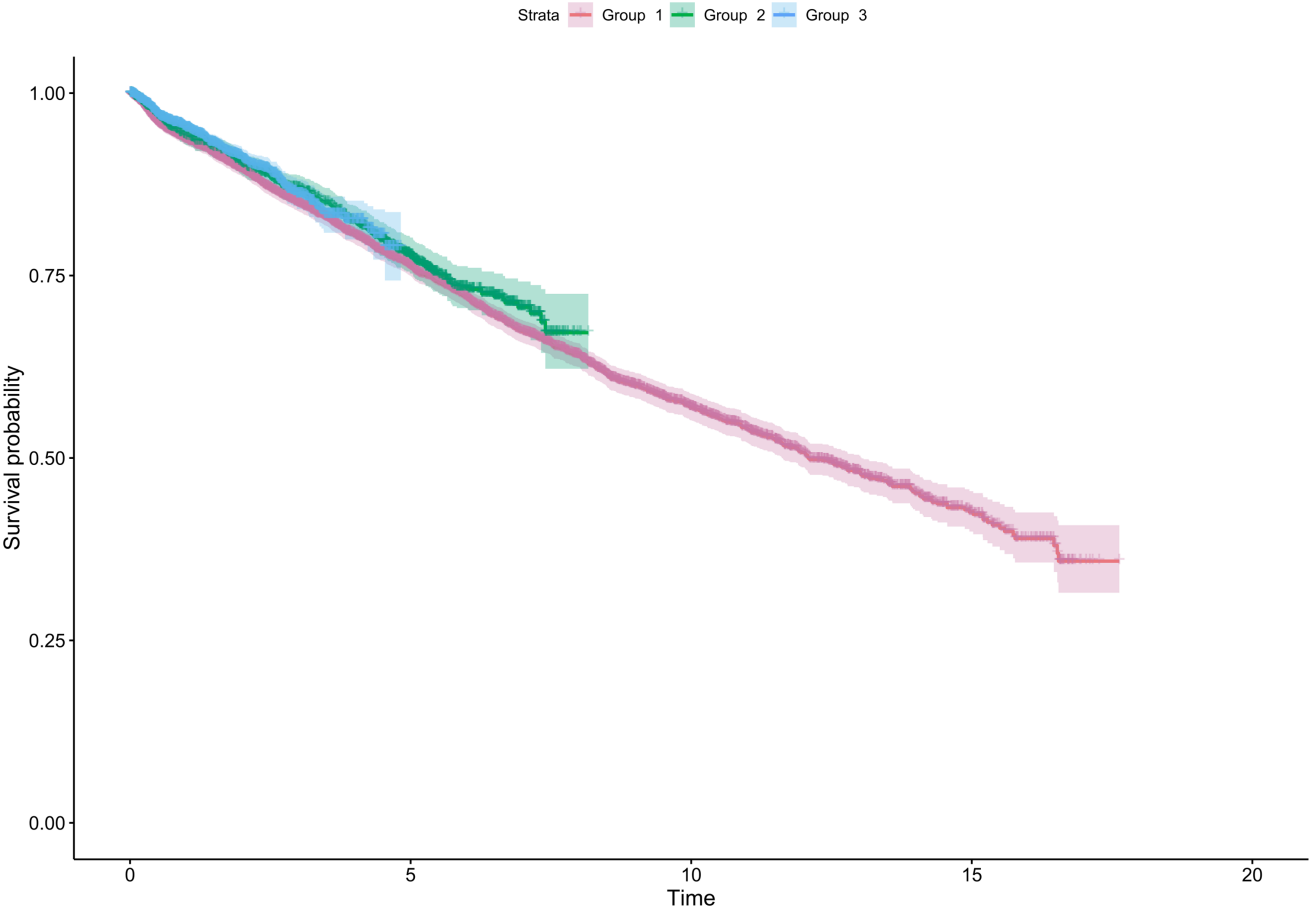
Kaplan–Meier graph showing the survival without untoward events of non-surgical root canal treatments with three different treatment methods from 0–17 years after initiation of the treatment. Curves with 95% confidence intervals, time in years, Group 1 –hand instruments, Group 2 –rotary NiTi instruments + PUI, Group 3 –Reciproc + PUI.

During the observation period (mean = 3.9 years [min. 0.0–max. 17.6 years]) 2,140 untoward events were registered in 9,938 included teeth. The Kaplan–Meier survival curves of the three observation groups without untoward events are shown in Fig. 1. The estimated five years survival rates without untoward events were 73.9% (95% CI [71.7%–76.1%]), 75.1% (95% CI [71.7%–78.0%]) and 78.4% (95% CI [75.1%–81.4%]) for study group 1, 2 and 3, respectively. The respective point estimates of the 1–5 years survival probabilities are additionally summarised in Table 2. Though not impressive, the differences between Group 1 and 3 were statistically significant (*p* = 0.006) in the Weibull regression model (Table 3). The decrease of the HR between Groups 1 and 3 was 20%. The survival curves show a nearly constant linear decline over time, while there is a difference between all curves during the first two to three years of the observation period (Fig. 1). Later on the curves of Group 2 and 3 cross each other. The Variables assessed are summarized in Table 3. Gender of the patient, number of intracanal dressings, root canal filling in the same session as canal preparation, treatment of vital teeth and previous experience of apicoectomies did not make a significant contribution to the modelling (all *p* > 0.05). In contrast, higher age of the patient and number of earlier NSRCTs reduce the survival without untoward events statistically significant (both *p* < 0.02). Treatment of premolars had a statistically significant lower HR (0.89 (95% CI [0.79–0.99]; *p* = 0.030) compared to treatment of molars and anterior teeth. Notably, a higher number of executed SPTs improved tooth survival without untoward events highly significant (*p* < 0.001).

## Discussion

Which concept for root canal treatment is the best? A recent review article reported that the heterogeneity and the limited number of clinical studies, as well as their insufficient case numbers for the purpose of meta-analyses prevent drawing reliable conclusions (Del Fabbro et al., 2018). The results of the present study show a statistically significant higher tooth survival rate without untoward events for endodontic treatments using reciprocating root canal instrumentation supplemented with PUI compared to hand instrumentation with syringe irrigation. Moreover, treatment of premolars and higher number of executed SPT have been identified as putative factors improving survival, whereas higher age and higher number of earlier NSRCTs in the same patient have been detected as factors reducing tooth survival without untoward events. To the best of our knowledge this is the first study that explores a 5-year clinically relevant outcome parameter of endodontic treatment performed with a recent treatment protocol including among others reciprocating instruments and PUI in a larger patient sample.

**Table 2:** The 1-, 2-, 3-, 4-, and 5-year estimated survival probabilities for each of the three treatment groups on the basis of the regression results.

	**Survival without untoward events**									
	1 year		2 years		3 years		4 years		5 years	
**Treatment Groups**	Survival probability in %	95% CI in %	Survival probability in %	95% CI in %	Survival probability in %	95% CI in %	Survival probability in %	95% CI in %	Survival probability in %	95% CI in %
Group 1	94.1	93.3–94.7	88.6	87.3–89.7	83.4	81.8–84.9	78.5	76.5–80.3	73.9	71.7–76.1
Group 2	94.4	93.5–95.2	89.1	87.5–90.5	84.1	81.9–86.1	79.5	76.6–82.0	75.1	71.7–78.0
Group 3	95.2	94.4–95.9	90.7	89.1–92.0	86.4	84.2–88.3	82.3	79.5–84.8	78.4	75.1–81.4

**Notes.**

Group 1 - hand instruments, Group 2 - rotary NiTi instruments + PUI, Group 3 - Reciproc + PUI.

### Tooth survival

In the present study we observed 5-years predicted survival rates after NSRCT without untoward events of 73.9%, 75.1% and 78.4% for hand instrumentation with syringe irrigation, rotary MF preparation with PUI, and reciprocating SF preparation with PUI, respectively. Tooth survival without untoward events after NSRCT has been described in several large-scale epidemiologic studies (Burry et al., 2016; Chen et al., 2007; Lazarski et al., 2001; Salehrabi & Rotstein, 2004). The highest survival rates without untoward events at an 8-years follow-up of 95.9%, 96.4%, and 96.0% for anterior teeth, premolars and molars, respectively, have been published by Salehrabi & Rotstein (2004). Burry et al. (2016) and Chen et al. (2007) have reported 5-years survival rates without untoward events of 92.0% and 89.7%, respectively. The study of Lazarski et al. (2001) has found a calculated 24-month survival rate without untoward events after NSRCT of 90.6%. In the present study the 5-year survival rate of 78.4% without untoward events for the most contemporary SF reciprocating technique with PUI is substantially lower compared to the latter studies. Because the aforementioned studies were epidemiologic studies, none of them has assessed varying root canal preparation strategies. In a meta-analysis by Ng, Mann & Gulabivala (2010) an estimated 2–3 years survival probability of 86% has been assessed, which is also higher than the 5-years rate of 78.4% of the present study for the most contemporary instruments. In the abovementioned meta-analysis (Ng, Mann & Gulabivala, 2010) however, survival rates were calculated without considering untoward events. With this in mind, a direct comparison between this meta-analysis and the present study is not possible, because it is reasonable to assume that survival rates considering untoward events might have been lower.

**Table 3:** Weibull regression modelling for factors affecting survival of teeth without untoward events after non-surgical root canal treatment.

**Variable**	**Hazard ratio**	**95% confidence interval**	***P*-value**
Total included sample size	***N = 9,920***		
Treatment Groups			
Group 1 (Hand instruments)	1		
Group 2 (rotary NiTi files + PUI)	0.95	0.84–1.08	0.437
Group 3 (Reciproc + PUI)	0.80	0.69–0.94	**0.006**
Root canal filling at the same appointment as canal preparation	1.15	1.00–1.31	0.053
Participants age (per year increase)	1.02	1.02–1.03	**<0.001**
Number of supportive periodontal treatments (per unit increase)	0.95	0.93–0.96	**<0.001**
Number of intracanal dressings (per unit increase)	1.01	0.98–1.06	0.387
Tooth treated = vital	0.94	0.86–1.02	0.141
Number of earlier NSRCTs (per unit increased)	1.03	1.01–1.06	**0.018**
Number of earlier surgical endodontic treatments (per unit increase)	1.10	0.95–1.28	0.195
Type of tooth			
Anterior teeth	1		
Premolars	0.89	0.79–0.99	**0.030**
Molars	1.00	0.89–1.11	0.944
Gender male	0.95	0.87–1.03	0.221

**Notes.**

PUI passive ultrasonic irrigation NSRCT non-surgical root canal treatment NiTi nickel titanium

Bold *p*-values indicate statistical significance of *p* < 0.05 in the Weibull regression model.

### Impact of technological changes on outcome of endodontic treatment

In the present study we explored three endodontic treatment regimens, reflecting two technological changes over a period of 17 years. Notably, the underlying treatment strategies are known, because changes in endodontic technology were documented. Rotary MF instruments with PUI, and reciprocating SF instruments with PUI showed increasing survival rates without untoward events of 75.1% and 78.4%, respectively, compared to hand instrumentation with basic disinfection methods that revealed survival rates of 73.9%. Reciprocating SF instruments combined with PUI showed statistically significant higher survival rates without untoward events compared to hand instrumentation. In contrast, no statistically significant differences between rotary instrumentation and hand instrumentation or rotary instrumentation and reciprocating instrumentation could be assessed. Fleming et al. (2010) have reported that the rate of untoward events after stainless steel hand instrumentation was statistically significant higher than with hand/rotary stainless steel and NiTi files, while the survival rate of teeth was comparable between these study groups. Another study evaluating periapical healing following endodontic treatment has shown statistically significant higher success rates in the NiTiR group compared to the group of stainless steel hand instruments (Cheung & Liu, 2009). Although these findings are partly in contrast to the present study, the differences can be explained by the fact that we have not included and cannot include periapical healing as an outcome parameter of endodontic treatment, because of the claims data character of the data base. Another study that has assessed the change from hand instruments to rotary instruments in combination with educational courses has shown that the calculated tooth survival rate—without consideration of untoward events though—increased statistically significant from 85% to 97% in post-education treatments (Koch et al., 2015). It is difficult to compare this study with the present study because education might have had a higher impact on reduced extraction rates than the technological change itself.

In the present study we observed higher survival rates without untoward events when recent treatment methods were applied. However, we cannot distinguish between the effect of modern MF rotary and SF reciprocating instruments for root canal preparation having a higher probability to prepare root canals more complete (Bartols, Robra & Walther, 2017; Zuolo, Carvalho & De-Deus, 2015) and a putative additional positive effect attributed to optimized disinfection through PUI. While *in-vitro* studies demonstrate a better cleanliness of root canals when using PUI (Caputa et al., 2019; Goodman et al., 1985; Haapasalo et al., 2010; Jensen et al., 1999), a prospective clinical study could not uncover any improvement by this method (Liang et al., 2013). A systematic review came to the conclusion, that the overall level of evidence on ultrasonic activation of endodontic irrigants is low, so that a clinical recommendation cannot be given (Balto, 2011).

In the present study there are several additional factors that might have influenced the treatment outcome. Apart from varying irrigation methods the irrigation solutions have been changed as well. A systematic review came to the conclusion, that the type of irrigant did not show an obvious trend on endodontic outcome and that further analyses were not possible because of insufficient data (Ng et al., 2008). Subsequently, with regard to periapical healing, a clinical study has reported a negative impact of Chlorhexidine on initial treatment cases and a positive impact of EDTA on endodontic retreatment cases (Ng, Mann & Gulabivala, 2011b), without any effect on tooth survival though (Ng, Mann & Gulabivala, 2011a). Thus, it is obvious that clinical studies remain contradictive as to varying irrigants. In addition, in the present study, obturation methods were partly changed from lateral to vertical compaction. While one study reported a positive impact of a vertical compaction technique, compared to lateral compaction on periapical healing (De Chevigny et al., 2008), other studies did not report any effect of the compaction method on periapical healing or on tooth survival (Ng, Mann & Gulabivala, 2011a; Ng, Mann & Gulabivala, 2011b).

Taken together, in the present study, a conclusion on the individual influence of the irrigation method or the obturation method on tooth survival without untoward events following root canal treatment cannot be drawn.

### Main predictors for survival without untoward events

In this more long-term study, the treatment of vital or non-vital teeth was not a factor influencing the survival of teeth after endodontic therapy. The influence of preoperative pulp status on treatment outcome is controversially discussed in the literature. Since bacterial infection is mostly associated with non-vital teeth, it seems to be likely that endodontic retreatment would be more often necessary due to incomplete disinfection during the first intervention resulting in persistent symptoms of the treated tooth. Indeed, non-vital teeth with apical lesions are reported to have lower success rates in periapical healing following endodontic treatment than vital teeth or non-vital teeth without apical lesions (Friedman & Mor, 2004; Ng et al., 2008). In contrast, a meta-analysis claimed comparable survival rates following endodontic treatment of teeth with or without apical lesions (Ng, Mann & Gulabivala, 2010) even though individual included studies were contradictory. In the present study we have not solely assessed tooth survival rates but tooth survival rates without untoward events. Although this additional parameter would explain lower survival rates, in our study we found comparable survival rates without untoward events in both vital and non-vital teeth. This finding is in accordance to the abovementioned meta-analysis.

Root canal filling performed in the same session as canal preparation turned out to be a factor that did not significantly influence eventless survival without untoward events. This observation seems to be in concordance to other published data. Most studies, including meta-analyses, show that treatment success rates are comparable between single- or multiple visit treatment (Wong, Zhang & Chu, 2014). However, to our knowledge there are no studies available reporting on tooth survival rates without untoward events following single- or multiple visit root canal treatment. Consequently, a direct comparison between our study and the other studies is not possible.

Further analyses showed (Table 1), that treatments with root canal preparation and root canal filling in one visit were significantly more often possible in Group 3 (25.2%) than in Group 1 (13.2%) and 2 (5.6%). This is consistent with another prospective clinical study of our workgroup in which the number of treatment sessions with SF Reciproc instruments tended to decrease compared to hand instruments in a general dental practitioner setting (Bartols et al., 2016b). Even if the treatment in as few sessions as possible is not a clinically relevant outcome measure, it is still a patient-friendly aim to be able to complete endodontic therapies as quickly as possible without sacrificing clinical outcome.

The impact of age on tooth survival following endodontic treatment has been controversially discussed. Our result that increased age of the patient is a factor reducing tooth survival without untoward events following root canal treatment is in accordance with other studies (Lazarski et al., 2001; Ng, Mann & Gulabivala, 2010). Although certain studies do not support this finding (Dammaschke et al., 2003; Nagasiri & Chitmongkolsuk, 2005), it should be noted that in these studies the case-numbers are rather limited, implying that differences might not have been detected.

In the present study, premolars showed a significantly lower loss probability (HR 0.89; *p* = 0.030) compared to anterior teeth and molars (both HR 1.0). While the last tooth in the dental arch appears to be associated with a relatively higher risk of loss after endodontic therapy (Ng, Mann & Gulabivala, 2010; Skupien et al., 2013), the risk of loss with respect to the tooth type is described inconsistently in the literature. While some studies identified a higher risk of loss in molars (Chen et al., 2007; Lazarski et al., 2001; Ng, Mann & Gulabivala, 2010), other studies found no differences between tooth types (Dammaschke et al., 2003; Ng, Mann & Gulabivala, 2011a; Skupien et al., 2013).

Moreover, also the number of earlier NSRCTs in the same patient was a significant factor in our study that was associated with a lower survival probability. This factor can be interpreted as indicating that patients with above-average endodontic treatment needs are likely to have greater overall dental treatment needs and are therefore at higher risk. Another study has shown that patients with more missing teeth have a higher risk of tooth loss in endodontically treated teeth (Skupien et al., 2013). As it was not possible to determine the number of missing teeth due to the data structure in the present study, the factor “number of earlier NSRCTs” was used as proxy for overall treatment need.

An interesting additional result is the correlation of SPT and tooth survival without untoward events following endodontic treatment. In the present study, cases that were subjected to periodontal aftercare showed statistically significant lower rates of untoward events. Despite the well-known reciprocal interaction between endodont and periodont, reflected by the fact that teeth with periodontal disease show high survival rates if additional endodontic treatment is necessary (Jaoui, Machtou & Ouhayoun, 1995), there is no study that has addressed the question whether preventive care, such as SPT, might have an impact on endodontic treatment outcome. Despite that, it is unclear if patients who seek SPT are not generally more interested in tooth preservation and therefore per se the probability of tooth preservation is higher. In any case, it must be pointed out that it is completely unknown whether SPT has any positive effect at all on endodontic therapy due to biological processes. Along this line, further studies are required to substantiate our finding and—if any—to uncover its putative significance.

### Influence of the operator on endodontic outcome

The influence of the operator on endodontic success is described in the literature especially for specialist vs. generalist treatments. One study found a higher success rate when endodontic therapy was performed by specialists (Alley et al., 2004) and a further study showed that the 10-year survival rate of molars was higher when the endodontic therapy was performed by specialists (Burry et al., 2016). Another study did not show any benefit of specialist therapy in endodontic treatment, but if additional root end resections were necessary after initial therapy, a higher survival rate was found when performed by specialists (Lazarski et al., 2001). However, a study with only a small sample size showed no differences in survival between specialist and generalist treatment (Tilashalski et al., 2004). A review also reported no significant influence of the operator on the survival of endodontically treated teeth (Ng, Mann & Gulabivala, 2010). In addition, Stoll, Betke & Stachniss (2005) found that the survival rate of endodontically treated teeth was not different when the therapy was performed by dentists or students under supervision. It is difficult to estimate the extent to which the dentist had an influence on the survival of the teeth examined in the present study. Due to the limitation of the database used, an exact allocation of the treatments to individual dentists is not possible in parts. However, certain homogeneity of the 49 dentists is given by the fact that no dentist with a specialist designation as an endodontist was involved in the therapy of the included cases. Furthermore, all dentists had a general dental treatment profile and none had more than four years of professional experience after graduating from university before working in the outpatient clinic.

### Limitations of the study

We are aware of the limitations of our study. These limitations apply for all similar endodontic studies because of the incomplete nature of the underlying claims data base, which do not contain individual patient-related information, such as general diseases, ethnic and socioeconomic background, treatment needs or habits, all putative confounding factors. This is also true for the present study. Moreover, whether e.g., a low recall might lead to response bias should be interpreted in light of previous studies. Interestingly, a review on endodontic studies has reported recall rates between 11–100% —with a median recall rate of 53% for initial treatments —and could not reveal any statistically significant influence of the recall rate on the treatment success rate (Ng et al., 2007). Therefore it is likely that the influence of the recall rate on treatment success might be overestimated.

In connection with the evaluation of the underlying routine (claims) data in this study, another limitation of the study became apparent. Due to the database structure, no reliable information on post-endodontic restoration was available. In various studies, it was found in particular that teeth with temporary restorations, amalgam restorations or glass ionomer cement fillings are at a higher probability of failure regarding fractures or survival (Dammaschke et al., 2013; Lazarski et al., 2001). Further studies also showed that crowns placed on endodontically treated teeth were associated with higher survival rates or higher resistance to fracture than post-endodontic restorations with fillings (Ng, Mann & Gulabivala, 2010; Ng, Mann & Gulabivala, 2011a; Salehrabi & Rotstein, 2004; Suksaphar et al., 2018). On the other hand, it was also shown that teeth with smaller composite restorations up to a maximum of two filled tooth surfaces and two proximal contacts have similar resistance to fracture as crowned teeth in premolars (Suksaphar et al., 2018). However, the problem that the post-endodontic restoration could not be included in the analysis due to the limitations of the database itself is also known from other endodontic studies with large scale secondary databases (Chen et al., 2007; Lin et al., 2014). Here, too, the evaluation of the parameter had to be dispensed with. It therefore remains speculative within the scope of the current study to what extent different restoration methods were used in the different study groups and ultimately have an influence on the survival rate of the examined teeth.

It should be noted that there is some statistical uncertainty about the superiority of treatment Group 3 over Group 1. This is due to the fact that the confidence intervals increase towards the end because of the shorter observation periods and then overlap in Groups 2 and 3. Nevertheless, the differences become significant and Group 3 is always superior to the other groups when considering the point estimators (see Table 2). In this context, the question of the clinical relevance of minor differences in survival probability should also be discussed. Since the differences between the observation groups are indeed small, there will be no perceptible improvement in the outcome of endodontic therapy at the level of a single care unit such as a dental practice due to the small number of cases. However, if the level of population-based care is considered, the joint effort of many dentists over time could lead to improved care for the general population. There will therefore be no perceptible effects for a single practitioner, but our study was not designed for that either. We understand this study rather as an observation of changing dental care that has taken place, in which there are indications that the use of more modern instruments is in all likelihood worthwhile.

## Conclusion

Within the limitations of the present study, we found some indications that the most recent endodontic treatment protocol, namely a combination of machine-driven SF reciprocating root canal instruments supplemented with PUI appears to be associated with higher estimated survival probabilities without untoward events compared to hand instrumentation with syringe irrigation. The effort of using more modern instruments seems to be justified against this background.

##  Supplemental Information

10.7717/peerj.8495/supp-1 Supplemental Information 1 Raw Claims Data used for analyses All descriptors for analyses as described in the Methods

## Additional Information and Declarations

### Data Availability

The following information was supplied regarding data availability: Data is available in a Supplementary File.
